# Pink Eccrine Chromhidrosis Induced by Excessive Consumption of an Energy Drink

**DOI:** 10.7759/cureus.71854

**Published:** 2024-10-19

**Authors:** Laetitia Van Houtte, Audrey Bulinckx, Pierre-Paul Roquet-Gravy

**Affiliations:** 1 Dermatology, Grand Hôpital de Charleroi, Charleroi, BEL

**Keywords:** chromonychia, eccrine chromhidrosis, energy drink, food dyes, pink sweat

## Abstract

Eccrine chromhidrosis is a rare condition characterised by the abnormal colouration of eccrine sweat, which may be triggered by endogenous or exogenous factors. We report the case of a 19-year-old male who was referred to us for persistent pink sweating for three months. Clinical examination and further investigations did not provide a clear diagnosis or arguments in favour of pseudochromhidrosis or apocrine chromhidrosis. Investigation of eating habits revealed an excessive consumption of a pink energy drink. The sweat normalised when consumption ceased, and chromhidrosis relapsed after the reintroduction of the drink. The diagnosis of eccrine chromhidrosis was retained. Eccrine chromhidrosis poses a diagnostic challenge, highlighting the importance of thorough patient history-taking, including lifestyle habits, and our case reveals the complex role of food additives present in energy drinks.

## Introduction

Chromhidrosis is an uncommon condition defined by the unusually coloured release of sweat, of which three subtypes have been identified: eccrine chromhidrosis, apocrine chromhidrosis and pseudochromhidrosis. Apocrine and eccrine chromhidrosis involve the emission of coloured sweat from the glands, unlike pseudochromhidrosis where colouration occurs on contact with the skin [1].

The causes of eccrine chromhidrosis may be endogenous, linked to underlying medical conditions, or exogenous, such as through the ingestion of dyes. Effective management involves treating the underlying causes [2]. We report the unique case of a young patient with eccrine chromhidrosis of exogenous origin, related to a dietary habit associated with the presence of food additives.

## Case presentation

**Figure 1:**
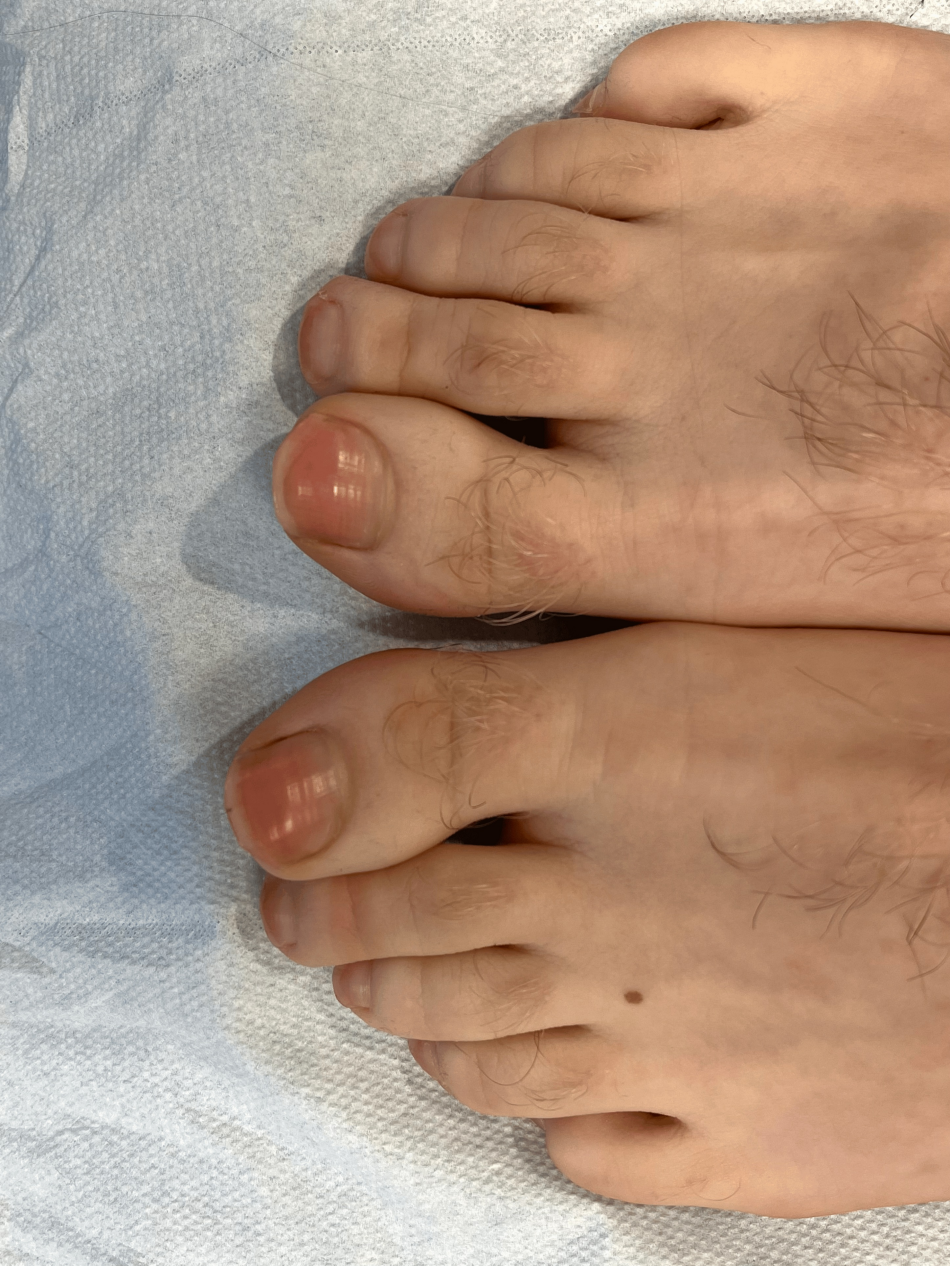
Pink chromonychia in the toenails

**Figure 2:**
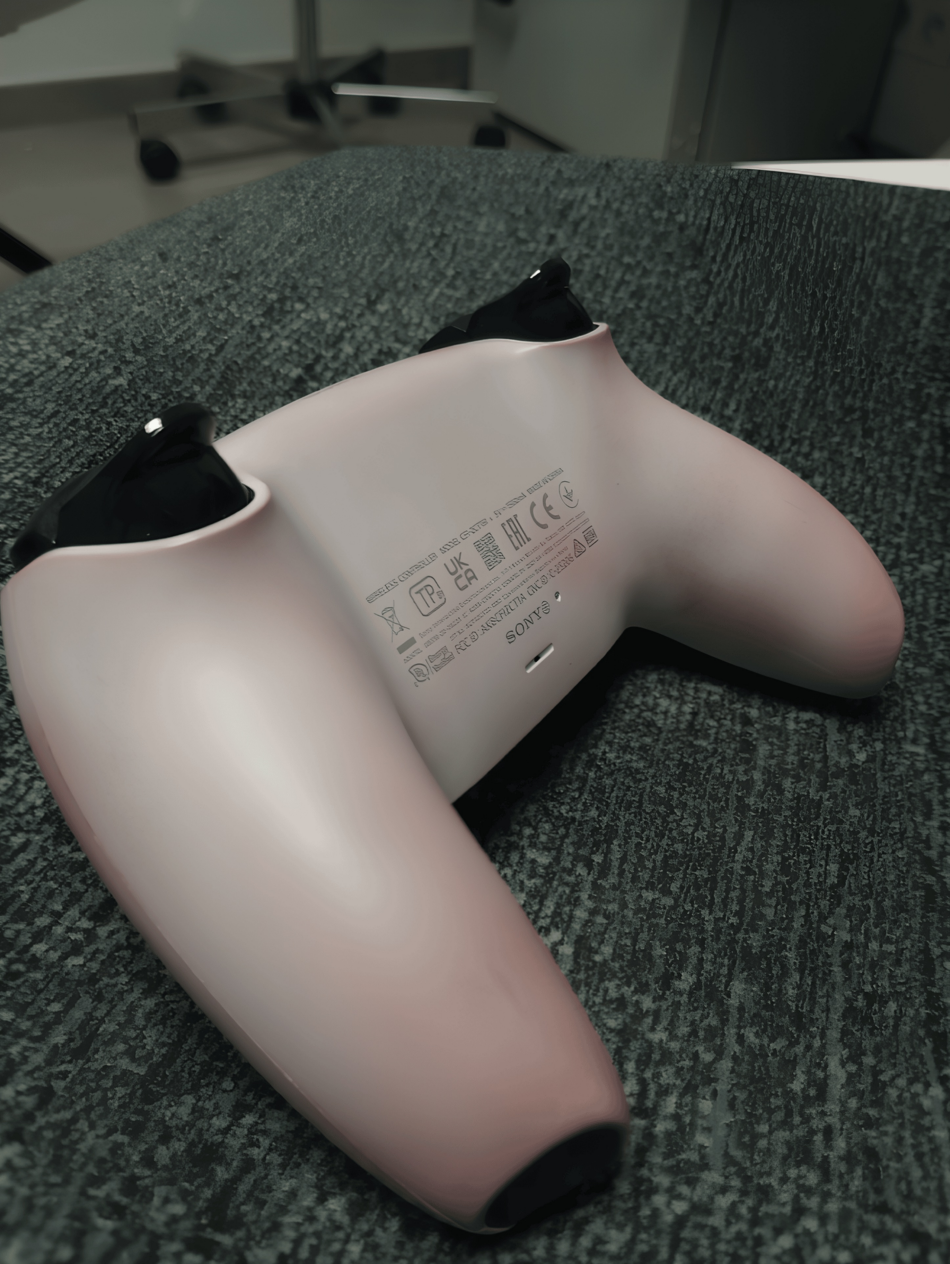
Pink discolouration of the PlayStation controller, initially white

**Figure 3:**
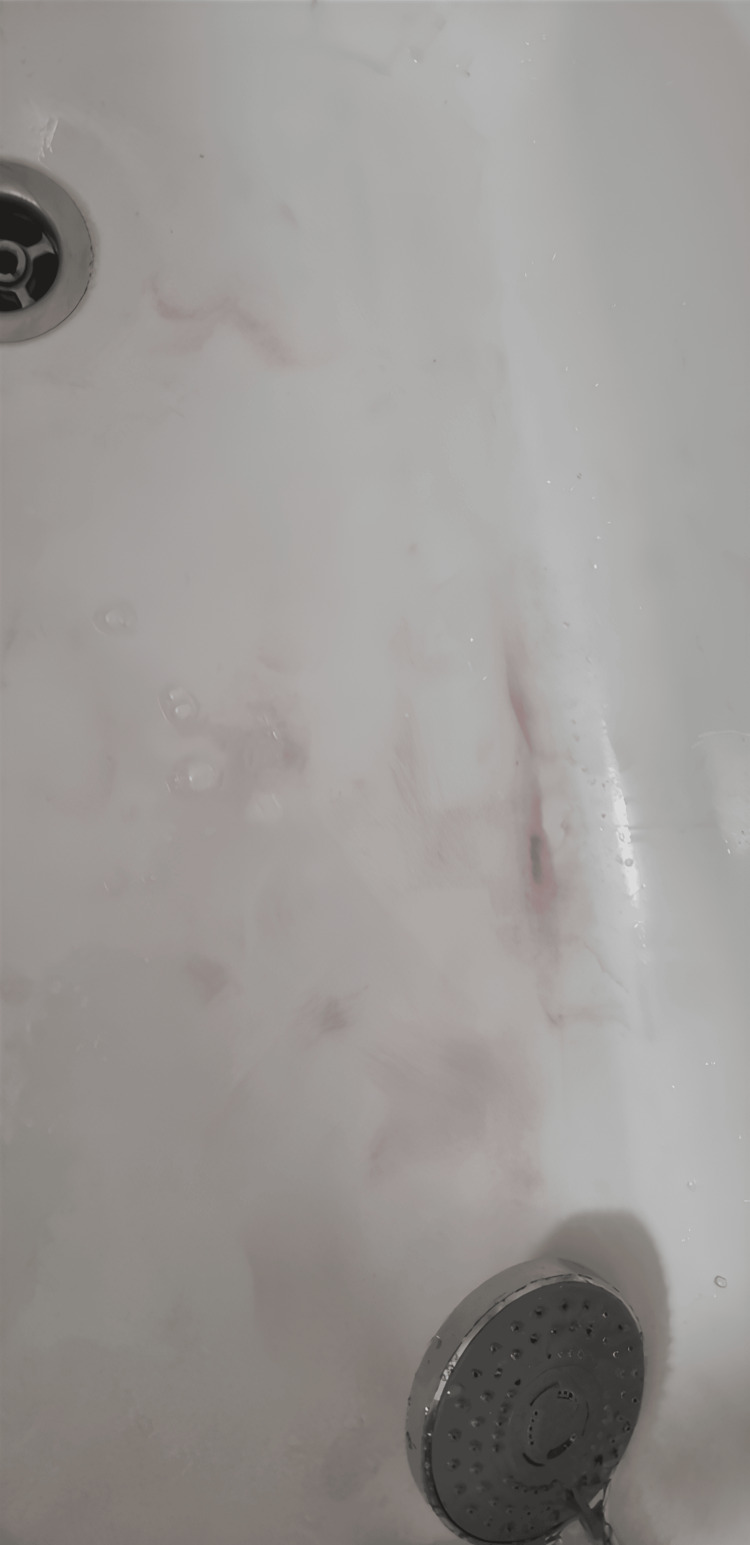
Pink discolouration of the bathwater

A young man, aged 19, who had trained as a pastry chef, was referred to our clinic by his general practitioner because of persistent pink perspiration for three months. The patient was in good health, with no chronic treatment nor significant medical or surgical history. He reported pink discolouration not only of his fingernails (Figure 1) and clothes but also of the colour of his bathwater and even on his PlayStation controller (Figures 2, 3).

He stated that his perspiration had no specific odour and that he did not regularly apply anything to his skin or clothes. Clinical examination revealed pink chromonychia of the toenails. The rest of the clinical examination was unremarkable. Urine and saliva were normal in colour. Examination under Wood's light did not reveal any fluorescence. A bacteriological skin smear was taken. The first diagnostic hypothesis was pseudochromhidrosis caused by *Serratia marcescens*, a chromogenic bacterium that colours sweat pink or red by releasing a pigment under specific conditions. Pending the results of bacteriological sampling, a trial treatment with erythromycin followed by ciprofloxacin was initiated, with no significant improvement in symptoms.

**Figure 4:**
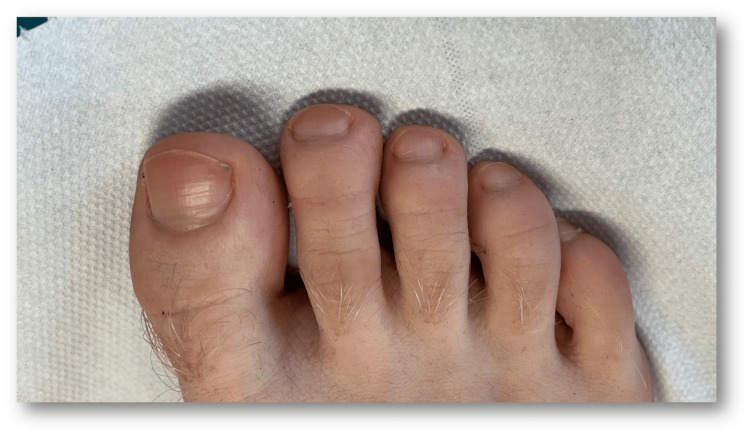
Nail discolouration after two weeks off the energy drink

Thereafter, skin biopsy for culture, as well as direct examination and culture of a nail sample, did not provide conclusive results. The bacteriological skin smear results also returned negative. The histology of the skin biopsy taken from the axilla was non-specific, providing no evidence of pseudochromhidrosis and revealing no lipofuscin granules in the apocrine glands. In the absence of an aetiology, further questioning, particularly about the patient's dietary habits, revealed an excessive consumption of a pink energy drink, which had been consumed daily for months. The diagnosis of eccrine chromhidrosis due to food colouring was therefore considered. When the drink was stopped, the patient's sweat returned to being colourless within two weeks (Figure 4).

The reintroduction test showed a recurrence of the pathological condition after two months of daily consumption.

## Discussion

Chromhidrosis is classified into three main subtypes, with distinct clinical manifestations depending on the mechanism involved.

Apocrine chromhidrosis generally appears after puberty, with preferential locations on the face, axilla, areola and ano-genital region due to the increased density of glands in these areas. This condition is associated with a greater concentration of lipofuscin granules in the glands, and their level of oxidation determines the colour excreted. Wood's light fluoresces yellow in yellow, green, and blue apocrine chromhidrosis [1].

In pseudochromhidrosis, sweat is coloured on contact with the skin following interaction with exogenous factors present on the skin, such as dyes, metals, chemicals or chromogenic bacteria. *Serratia marcescens* induces a pink-red discolouration of sweat through the emission of a pigment called prodigiosin, generated under favourable conditions. Appropriate antibiotic treatment, although not codified, leads to rapid resolution of the condition [3].

Finally, eccrine chromhidrosis is a generalised disorder involving the excretion of water-soluble pigments in sweat [4]. Eccrine glands are widely distributed throughout the body, with a greater concentration on the palms and soles [5]. The causes of eccrine chromhidrosis are diverse and can be classified into two categories: exogenous and endogenous. Exogenous causes include ingestion of medicines containing quinine [2], exposure to metals such as copper [6] and consumption of dyes such as tartrazine [7], food dyes [2,4] or natural plant pigments such as betalains found in certain fruits such as pitaya [8]. Endogenous factors are mainly associated with hyperbilirubinemia, which manifests as greenish sweat, mainly on the palms and soles, sometimes with a clinical appearance of dyshidrotic eczema [5]. Uremic frost, a rare complication of severe renal failure, results from hyperuricemia and is characterised by white-to-off-white, crystalline sweat, especially present in eccrine and hairy sweat areas such as the face, neck, scalp, forearms and torso [9].

Two potentially responsible water-soluble dyes were identified in our patient's energy drink: carminic acid (E120) and anthocyanin (E163). Mass spectrometry could have helped to determine the agent responsible but was not carried out for our patient [4]. Another case in the literature has raised the possibility of the imputability of anthocyanin in eccrine chromhidrosis, but this has not been confirmed [2].

Our patient's chromonychia almost completely disappeared 15 days after stopping the energy drink. Renewal of a toenail takes an average of 12-18 months [10]. This suggests that the nail colouration was due to contamination of the surface layer of the nail plate by the surrounding sweat, thus rejecting the idea that pigment was present in the matrix. In addition, our patient's nail colouration followed the shape of the proximal nail fold, thus arguing for an external origin of the dyschromia [11].

## Conclusions

Aetiological research and determination of the underlying cause of chromhidrosis are crucial in defining an appropriate therapeutic strategy and rapid resolution of the symptoms, as demonstrated by our case report. This case of pink eccrine chromhidrosis associated with the overconsumption of an energy drink highlights the diagnostic challenge of chromhidrosis and the importance of thorough patient history-taking, particularly regarding lifestyle habits. It underscores the complex role of exogenous factors, such as food additives, in the development of this rare condition.
